# Osteoma Cutis Associated with Nevus Sebaceus: Case Report and Review of Cutaneous Osteoma-associated Skin Tumors (COASTs)

**DOI:** 10.7759/cureus.4959

**Published:** 2019-06-20

**Authors:** Martin J Azzam, Bryce D Beutler, Antoanella Calame, Christof P Erickson, Philip R Cohen

**Affiliations:** 1 Internal Medicine, University of Nevada, Reno School of Medicine, Reno, USA; 2 Dermatology, Compass Dermatopathology, Inc., San Diego, USA; 3 Dermatology, San Diego Family Dermatology, National City, USA

**Keywords:** cutis, hamartoma, nevus, osteoma, primary, sebaceous, sebaceus, secondary, skin, tumor

## Abstract

Osteoma cutis is a benign cutaneous lesion characterized by the presence of bone within the dermis or subcutaneous fat. It most often develops in association with other skin lesions such as cutaneous tumors. Nevus sebaceus is a benign hamartoma of the skin that is composed of epidermal and dermal components. It most commonly appears on the scalp and may give rise to either benign or malignant secondary neoplasms. The clinical and pathologic features of a 36-year-old man with a nevus sebaceus and associated osteoma cutis are described. In addition, osteoma cutis-associated neoplasms are reviewed. Secondary osteoma cutis has been observed with both benign and malignant neoplasms as well as various non-neoplastic skin conditions. However, to the best of our knowledge, osteoma cutis has not previously been described in association with nevus sebaceus. Nevus sebaceus can now be added to the list of cutaneous osteoma-associated skin tumors (COASTs).

## Introduction

Osteoma cutis, the heterotropic deposition of bone within the dermis, may either present rarely as a primary skin lesion or more commonly secondary to another cutaneous condition. The pathogenesis of osteoma cutis has yet to be established. However, a derangement of the intercellular signaling mechanisms resulting in the aberrant proliferation of dermal fibroblasts with subsequent metaplastic transformation to bony tissue has been postulated [1].

Nevus sebaceus is a benign neoplasm representing a congenital malformation of the pilosebaceous follicular unit. It is a hamartomatous growth that most commonly forms on the scalp, but may also appear on the forehead, face, or neck. These lesions are typically subtle in appearance at birth and during childhood; they usually become more prominent following puberty secondary to hormonal changes that induce a growth phase of the tumor. Nevus sebaceus may also develop various benign or malignant secondary neoplasms during adulthood [2].

A man with a progressively enlarging scalp tumor of congenital onset is described. Biopsy of the neoplasm showed a nevus sebaceus; secondary osteoma cutis was observed in the excision specimen. Other neoplasms associated with incidental osteoma cutis are also reviewed.

## Case presentation

A 36-year-old Filipino man presented with a lesion on his scalp that had been present since birth, but had been progressively enlarging over the past several years. The patient noted that the lesion increased in size during puberty. However, between adolescence and age 30 years, neither the size nor the appearance of the lesion had changed.

**Figure 1:**
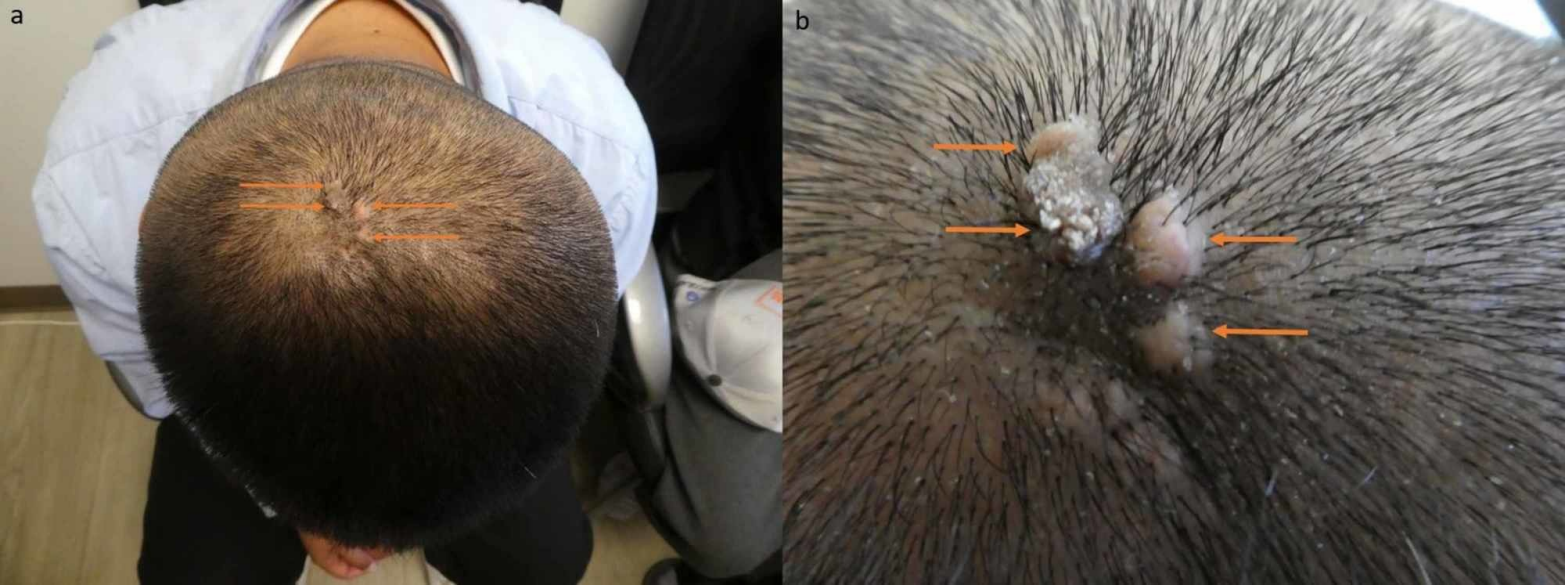
Clinical presentation of nevus sebaceus with associated osteoma cutis on the scalp of a 36-year-old Filipino man. Distant (a) and closer (b) views of secondary osteoma cutis associated with nevus sebaceus on the central scalp of a 36-year-old Filipino man. The tumor consists of multiple individual and confluent flesh-colored and brown papules and nodules (orange arrows) on the scalp.

Cutaneous examination showed multiple individual and confluent flesh-colored and brown papules and nodules covering a total body surface area of 27 mm x 15 mm on the patient’s scalp. Each nodule measured approximately four to six millimeters in diameter. Hair was absent in the area occupied by the tumor (Figure 1). A punch biopsy was performed.

**Figure 2:**
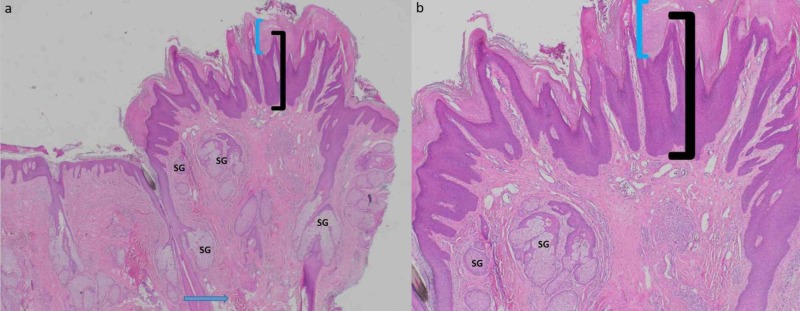
Pathology presentation of nevus sebaceus on the scalp of a 36-year-old Filipino man: initial biopsy. Distant (a) and closer (b) magnification views show a nevus sebaceus consisting of hyperkeratosis (thickening of the stratum corneum as shown between the blue bracket), acanthosis (thickening of the epidermis as shown between the black bracket), and papillomatosis in the epidermis. In addition, there are an increased number of apocrine glands (blue arrow) and enlarged sebaceous glands (SG) in the dermis. [Hematoxylin and eosin: a, x4; b, x10].

Microscopic examination demonstrated hyperkeratosis, papillomatosis, and acanthosis with an increased number of sebaceous and apocrine glands in the dermis. These findings established a diagnosis of nevus sebaceus (Figure 2).

**Figure 3:**
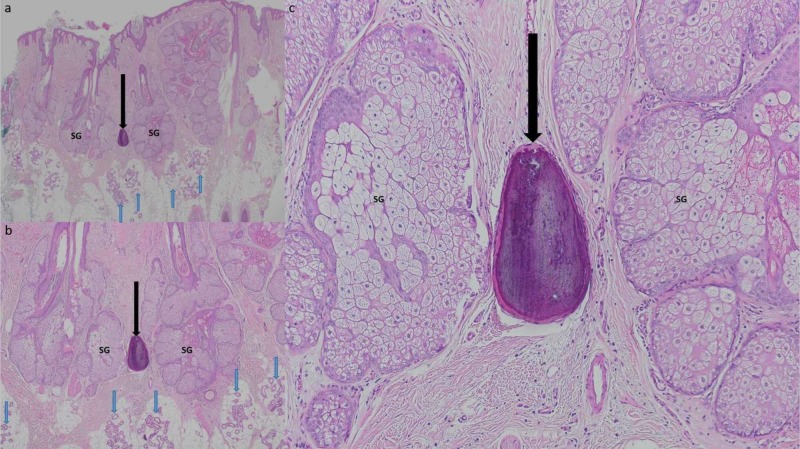
Pathology presentation of nevus sebaceus with associated osteoma cutis on the scalp of a 36-year-old Filipino man: excision specimen. Distant (a) and closer (b and c) magnification views of secondary osteoma cutis associated with nevus sebaceus. There is hyperkeratosis, acanthosis, and papillomatosis in the epidermis. In the dermis, there are an increased number of apocrine glands (blue arrows) and enlarged sebaceus glands (SG); in addition, osteoma cutis (black arrow pointing at bone) is also present. [Hematoxylin and eosin: a, x2; b, x10; c, x40].

The residual tumor was completely excised. A layered closure was used to repair the wound defect. Evaluation of the excision specimen showed the same pathology changes noted in the biopsy. In addition, ossified bone was present in the dermis (Figure 3).

Correlation of the clinical presentation and pathologic findings established a diagnosis of nevus sebaceus associated with secondary osteoma cutis. The surgical site healed. The patient was assured of the benign nature of the lesion. He agreed to return for periodic routine follow-up visits.

## Discussion

Osteoma cutis is the formation of bone within the skin. It can occur as either a distinct dermatologic entity (primary osteoma cutis) or in association with other conditions (secondary osteoma cutis). It can also be further classified into various subtypes: plate-like, miliary, isolated, and disseminated osteoma cutis [3]. To the best of our knowledge, our patient is the first individual to present with secondary osteoma cutis arising in association with nevus sebaceus.

Osteoma cutis typically presents as a single, stone-hard white dermal and/or subcutaneous papule or nodule. In some individuals, multiple lesions may be present. Osteoma cutis is most often asymptomatic, but is occasionally painful. The scalp, face, and extremities are common sites [4]. Confirmation of the suspected osteoma cutis is usually established following microscopic examination after the lesion has been biopsied or removed [3]. Interestingly, asymptomatic osteoma cutis is occasionally discovered as an incidental finding on radiography [5].

Osteoma cutis has been associated with numerous cutaneous lesions and systemic conditions. These include hereditary syndromes of heterotropic bone formation or tissue deposition (Albright hereditary osteodystrophy, dermatomyositis, fibrodysplasia ossificans progressiva, Gardner’s syndrome, scleroderma, and systemic lupus erythematosus), inflammatory conditions (acne), infections (folliculitis and syphilis), and trauma (myositis ossificans and post-traumatic scars) [6-9]. Cutaneous osteoma-associated skin tumors (COASTs) are listed in Table 1 [6, 10].

**Table 1:** Cutaneous osteoma-associated skin tumors (COASTs).

Condition	Reference
Actinic keratosis	[6]
Atypical fibroxanthoma	[6]
Basal cell carcinoma	[10]
Chondroma	[6]
Chondroid syringoma	[6]
Desmoid tumor	[6]
Dermatofibroma	[6]
Epidermal nevus	[6]
Hemangioma	[6]
Infundibular cyst	[6]
Lipoma	[6]
Melanocytic nevus	[6]
Melanoma	[6]
Neurilemmoma	[6]
Trichoepithelioma	[6]
Trichofolliculoma	[6]

Microscopic examination is usually required to establish a diagnosis of osteoma cutis. Pathologic features include bony trabeculae containing osteoblasts and osteocytes in the dermis, subcutaneous fat, or both. Laboratory studies that may be used in the evaluation of osseous pathology--such as a complete blood count and serum chemistries (including vitamin D level, calcium, and alkaline phosphatase)--are typically normal [3].

The pathogenesis of osteoma cutis has yet to be definitively established, but a derangement of an inflammatory response is a speculated hypothesis. Indeed, many of the conditions associated with secondary osteoma cutis--including autoimmune disorders, hereditary syndromes of heterotropism, and neoplasms--are characterized by inflammation and dysfunctional intercellular signaling. It has been suggested that an inappropriate secretion of inflammatory cytokines leads to differentiation of dermal fibroblasts into metaplastic osteoblasts, which then result in the deposition of bone in the dermis and subcutaneous fat [1]. In addition, other investigators have postulated that primary osteoma cutis is the result of aberrant migration of normally differentiating mesenchymal cells; however, this theory has yet to be observed in vivo [3].

Nevus sebaceus, also referred to as “nevus sebaceus of Jadassohn” or “organoid nevus,” is a congenital hamartoma that was first described in 1895 by the German dermatologist Josef Jadassohn [11]. It is a hamartoma of the follicular, sebaceous, and apocrine components of the epidermis as well as the connective tissue of the dermis. Lesions are often associated with epidermal hyperplasia [12].

The diagnosis of nevus sebaceus is established based on its distinct clinical features. Lesions arise during childhood or adolescence and present as single, smooth, oval-shaped, yellow patches [3]. The most frequently affected site is the scalp. However, nevus sebaceus has also been described on the face and neck [13].

Cutaneous conditions associated with nevus sebaceus include both benign tumors and malignant neoplasms (Table 2) [14-15]. In most individuals, however, nevus sebaceus exists independent of any other skin lesions. In our patient, the nevus sebaceus was not associated with a benign tumor or malignant neoplasm.

**Table 2:** Cutaneous tumors associated with nevus sebaceus.

Tumor	Reference
Benign	
Eccrine poroma	[14]
Syringocystadenoma papilliferum	[14]
Trichilemmoma	[14]
Trichoblastoma	[15]
Malignant	
Apocrine carcinoma	[15]
Basal cell carcinoma	[14-15]
Eccrine carcinoma	[14]
Sebaceous carcinoma	[15]
Squamous cell carcinoma	[15]

Nevus sebaceus may occur as a manifestation of systemic syndromes. Nevus sebaceus syndrome is characterized by various brain, eye, and bone disorders as well as the presence of a large nevus sebaceus. Phakomatosis pigmentokeratotica is another systemic condition that presents with neurologic deficits and speckled lentiginous nevi with an associated nevus sebaceus. Hemiparesis, developmental delay, seizures, and other structural abnormalities within the brain may be present in both nevus sebaceus syndrome and phakomatosis pigmentokeratotica [16].

Nevus sebaceus are benign congenital skin lesions and are usually diagnosed clinically. However, histologic examination may be required if rapid growth is noted or if there are changes in the clinical features of the lesion that raise suspicion for malignancy. Nevus sebaceus is characterized by papillomatous hyperplasia of the epidermis and a proliferation of not only sebaceous glands but also apocrine glands in the dermis [14].

The natural history of a nevus sebaceus is divided into three stages. The first stage typically occurs during childhood and is characterized by the presence of a yellow-colored or pink-colored macule or patch. The lesion is stable in size throughout childhood, as the sebaceous glands have yet to mature in the absence of hormonal stimuli.

The second stage of nevus sebaceus occurs during adolescence. Hormonal influences on the sebaceous and apocrine glands induce thickening of the lesion. Dehydroepiandrosterone and testosterone are thought to represent the key mediators in this process.

The third stage of nevus sebaceus may occur when the individual reaches middle-age. It is characterized by growth and differentiation of another tumor originating from the previously quiescent lesion. The third stage does not occur in all individuals. Indeed, most nevus sebaceus rarely progress beyond the second stage [17].

Nevus sebaceus is often treated by surgical excision [10, 18]. Confirmation of complete tumor removal can be established either by using Mohs micrographic surgery and examination of the margins during the procedure or by processing the excised tissue for routine pathology evaluation. The depth of the excision should be to the level of the subcutaneous fat in order to minimize the risk of recurrence.

In the absence of a suspected nevus sebaceus-associated neoplasm, reassurance and monitoring of the tumor represent a reasonable alternative to resection. In addition, alternative treatment options that have been described for nevus sebaceus include carbon dioxide laser, dermabrasion, and photodynamic therapy [19]. However, as these modalities may not completely remove the lesion, the risk of recurrence persists.

The association of osteoma cutis and nevus sebaceus is unique. In our case, osteoma cutis was discovered as an incidental finding when our patient’s nevus sebaceus was excised. Henceforth, osteoma cutis can be added to the conditions associated with nevus sebaceus and nevus sebaceus can be added to the list of COASTs.

## Conclusions

Osteoma cutis is the development of bone within the skin; it can occur as either a primary lesion or secondary to another etiology, including inflammatory conditions, tumors, infections, and trauma. Nevus sebaceus is a benign hamartoma of the skin that is composed of epidermal and dermal components; it most commonly appears on the scalp and may give rise to either benign or malignant secondary neoplasms. Henceforth, osteoma cutis can be added to conditions associated with nevus sebaceus and nevus sebaceus can be added to the list of COASTs.
